# Chronic obstructive pulmonary disease mortality trends in Spain, 1980-2020

**DOI:** 10.4178/epih.e2023036

**Published:** 2023-03-18

**Authors:** Lucia Cayuela, José Luis López-Campos, Anna Michela Gaeta, Rocio Reinoso-Arija, Aurelio Cayuela

**Affiliations:** 1 Department of Internal Medicine, Hospital Severo Ochoa, Leganés, Spain; 2 Unidad Médico-Quirúrgica de Enfermedades Respiratorias, Instituto de Biomedicina de Sevilla (IBiS), Hospital Universitario Virgen del Rocío/Universidad de Sevilla, Sevilla, Spain; 3 Centro de Investigación Biomédica en Red de Enfermedades Respiratorias (CIBERES), Instituto de Salud Carlos III, Madrid, Spain; 4 Pneumology Service, Hospital Severo Ochoa, Leganés, Spain; 5 Unit of Public Health, Prevention and Health Promotion, South Seville Health Management Area, Seville, Spain

**Keywords:** Chronic obstructive pulmonary disease, Mortality, Epidemiology

## Abstract

**OBJECTIVES:**

In Spain, there has been a recent increase in the mortality rate for chronic obstructive pulmonary disease (COPD) in younger women. This study aimed to analyze trends in the COPD mortality rate in Spain from 1980 to 2020, evaluating any differences between genders and age groups.

**METHODS:**

Death certificates and mid-year population data were obtained from the Spanish National Institute of Statistics. For both genders, age group-specific and standardized (overall and truncated) rates were calculated by the direct method using the world standard population. The data were analyzed using the joinpoint regression method.

**RESULTS:**

In both men and women, the number of COPD deaths increased from 1980 to 1999 (average annual increase of 7% in men and 4% in women), while from 1999 onwards, deaths decreased by -1.0% per year in both genders. In women, there was a significant final period of increase in the 55-59 to 70-74 age groups and a slowing of the decline in the over 75 age group. Additionally, an increase in mortality for the truncated rates was observed for women between 2006 and 2020. In men under 70 years of age, there was an initial period in which death rates remained stable or significantly increased, followed by a period in which they decreased significantly.

**CONCLUSIONS:**

Our study shows age and gender differences in COPD mortality trends in Spain. Although the data show a downward trend, we have identified a worrying increase in the truncated rates in women for the last few years.

## INTRODUCTION

Chronic obstructive pulmonary disease (COPD) is a leading cause of disability and mortality worldwide, accounting for 212.3 million prevalent cases, 3.3 million deaths, and 74.4 million disability-adjusted life years in 2019 [1]. However, findings from studies over the past decades indicate that mortality from COPD has been declining worldwide since the 1990s [2]. In the period from 1995 to 2017, COPD mortality rates, in both genders, decreased or remained stable in Latin America and the Caribbean, Asia, and Oceania, although an increase was observed in Australian and Cuban women [3]. In the United States, the age-standardized mortality rate (ASMR) decreased for COPD among men from 1999 to 2019, but remained higher in men than in women. In women, although the overall rates showed no significant changes, they increased in some geographical subgroups [4].

In the European Union, most of the trends observed in both men and women have shown a favorable decline in COPD mortality rates, although not in a uniform way across Europe. In fact, 12 countries showed decreasing trends for men and 8 for women, while other countries showed varying increasing and decreasing trends over the period. Meanwhile, 4 countries showed final increasing trends for men (Belgium, Bulgaria, Greece, and Slovenia) and 3 for women (Bulgaria, Estonia, and Greece) in COPD mortality over the most recent periods assessed [5]. In addition, the latest joinpoint analyses have highlighted a recent, unexpected turn in the trends since 2014, with moderate increases in COPD mortality in some countries for both genders, including some of the largest Western countries, such as Italy, Germany, and the United Kingdom [6].

In Spain, studies have suggested that COPD mortality rates have been decreasing since the 1990s, especially among men [7,8], while in younger women (35-64 years), a recent increase in mortality rates has been observed [6]. Interestingly, previous studies on COPD mortality trends in Spain were based directly on the ASMR. However, the ASMR, being essentially a weighted summary measure, tends to reflect trends observed in older age groups and fails to describe differences between generations. Therefore, an updated analysis is needed to analyze all age groups, and to discriminate those that contain the younger population. Consequently, the aim of this study was to provide an updated view of the temporal trends in COPD mortality rates in Spain, with an emphasis on gender and age differences.

## MATERIALS AND METHODS

Data on population and COPD death records (International Classification of Diseases codes 490-492 and 494-496 for the ninth edition, and codes J40-J44, J47 for the 10th edition) were retrieved from the National Institute of Statistics for the study period (1980-2020). Intercensal populations were estimated on July 1 of each year based on the official census information. Age-specific rates were calculated for each 5-year period of age (from < 5 to ≥ 85 years), gender, and calendar year by the direct method, using the world standard population [10], at all ages and truncated at 35-64 years. These were expressed as rates/100,000 person-years.

To identify significant changes in mortality trends, we created joinpoint regression models and estimated annual percent changes (APCs) for each segment identified by the models. To quantify trends over the whole period, we computed the average APC as a geometrically weighted average of the various APCs from the joinpoint regression analysis, with weights being equivalent to the length of each segment during the specified time interval [11]. This represented a summary measure of the trend over the study period (1980-2020). If an average APC lay entirely within a single joinpoint segment, the average APC was equal to the APC for that segment.

At most, 5 joinpoints were allowed in the analysis. The minimum intervals until the extremes of the observation periods was established at 2 years for both the beginning and the end of the periods. An associated p-value < 0.05 represented the likelihood that the APC or average APC was significantly different from 0. In describing the trend analysis results, the terms “increase” or “decrease” indicate statistical significance (p< 0.05), while nonsignificant results are reported as “stable.” We used the software’s pairwise comparison option to verify whether trends were parallel for both genders [12]. All analyses were performed using the Joinpoint Regression version 4.9.1.0 (National Cancer Institute, Bethesda, MD, USA).

### Ethics statement

Because the data extracted from the National Institute of Statistics were anonymized, following the principles of good clinical practice and the Declaration of Helsinki, the participants were not identified, and no personal information was accessed; thus, this study did not require patient consent or approval from the ethics committee.

## RESULTS

Between 1980 to 2020, there were 552,174 deaths in Spain due to COPD. Of this total, 415,930 deaths occurred in men, with a man-to-woman ratio of 3.1. The lowest number of deaths was observed in 1980, with 6,379 deaths, and the highest was in 1999, with 17,992. For both men and women, the number of deaths increased during the period from 1980 to 1999 (average annual increase of 7% for men and 4% for women) and, from 1999 onwards, deaths decreased by -1.0% per year for both genders. The man-to-woman ratio increased during the period from 1980 to 2006 (from 2.0 in 1980 to 3.6 in 2006), after which the gender gap narrowed, and the man-to-woman ratio dropped to 3.0 in 2020.

**Figure 1.**
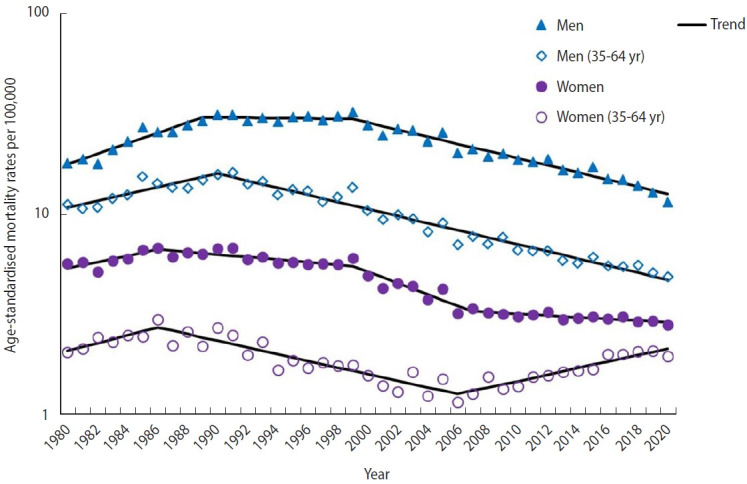
Time trends in age-standardized mortality rates (overall and truncated 35-64 years) from chronic obstructive pulmonary disease in men and women.

The ASMRs (all ages) due to COPD in men and women (1980-2020) are illustrated in Figure 1 and summarized in Table 1. The ASMRs for all ages in both men and women decreased significantly over the study period (from 18.02 to 11.48; average APC, -0.9% in men and from 5.71 to 2.82; average APC: -1.5% in women). Joinpoint analysis identified 3 periods in men (Figure 1 and Table 1): an initial period (1980-1989) in which rates increased significantly (APC, 6.1%), a second period in which rates remained stable (1989-1999) and a final period in which rates decreased significantly (1999-2020; APC, -4.0). In women (Figure 1 and Table 1), 4 time periods were observed: an initial period (1980-1986) in which rates increased significantly (APC, 3.7%) followed by 3 periods of significant decline, albeit at different rates (1986-1999; APC, -1.5%; 1999-2007; APC, -6.3%; and 2007-2020, APC, -0.9%). Accordingly, the man-to-woman ratio in the ASMR (all ages) increased from 1980 to 2006 (from 3.2 in 1980 to 6.3 in 2006), after which the gender gap narrowed, with the man-to-woman ratio decreasing to 4.1 in 2020.

During the study period, the truncated ASMR (35-64 years) showed a significant decrease only for men (APC, -2.1%; Figure 1 and Table 1). The joinpoint analysis identified 3 periods of significant changes in men (Figure 1 and Table 1): after an initial period with a significant increase (1980-1990; APC, 4.0%), the rates decreased significantly (1990-2020; APC, -4.0%). In women, there was an initial period with an increase (1980-1986; APC, 4.6%), a second period with a decrease (1986-2006; APC, -3.8%), and a final period with an increase (2006-2020; APC, 3.8%). Accordingly, the man-to-woman ratio for the truncated ASMR increased from 1980 to 1999 (from 5.4 in 1980 to 7.7 in 1999), after which the gender gap narrowed, with the man/woman ratio decreasing to 2.5 in 2020.

**Figure 2.**
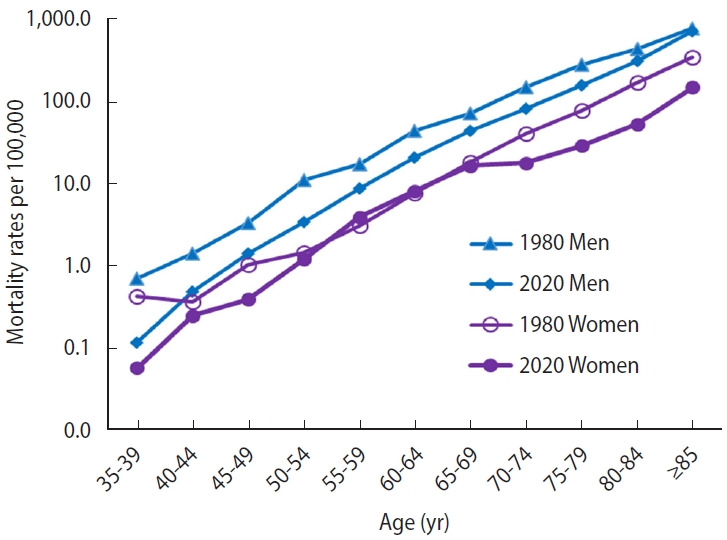
Mortality curves of mortality rates by age and gender in 5-year age groups for men and women in Spain in 1980 and 2020.

The specific mortality rates for 5-year age groups are shown in Figure 2. These trends were exponential with increasing age. The ASMRs by age groups are shown in Table 1, with the results of the joinpoint analysis in men and women, respectively. In men (Table 1), during the study period, there was a significant decrease in rates in all age groups under 80 years, while the rates remained stable in the 80-84 age and 85+ age groups. Joinpoint analysis in men under 70 years of age showed 2 periods: an initial period in which rates remained stable or showed a significant increase, followed by a period in which rates decreased significantly. Above the age of 70 years, a second period of stabilization or significant increase in rates was followed by a third period of significant decrease. For those over 80 years of age, there was a fourth period from 2015/2017 onwards, in which the decline observed in the previous period increased significantly and sharply.

In women, the rates by age groups (Table 1) remained stable in the age groups of 50-54 years, 55-59 years, 60-64 years, and 65-69 years, while the rest of the age groups showed significant decreases. The joinpoint analysis showed, since the early 2000s, a significant final period of increase in the 55-59 age to 70-74 age groups and a slowing of the decline in the over 75 age group.

## DISCUSSION

This study completed, updated, and analyzed mortality trends in Spain through 2020. Our data show, for the first time in the country, an increase in mortality in the truncated rates of women, which contradicts the traditional downward trend reported in previous analyses. Despite the decline in COPD mortality rates observed in many countries, due to population growth and aging, the actual number of COPD deaths has increased, or at least stabilized, in most of them [3]. The number of deaths caused by COPD increased from 2.5 million in 1990 to 3.3 million in 2019, with East Asia, South Asia, and Western Europe being the regions with the highest number of deaths in 2019 [1]. In Spain, in contrast to those countries, our results show that the decline in COPD mortality rates has been mirrored by a reduction in the number of deaths since 1999 (-1.0% per year in both men and women).

Our results are consistent with what has been observed in other countries, showing that although mortality rates have remained higher among men, there is evidence of a narrowing of the gender gap since the early 2000s, especially in truncated rates (35-64 years). Despite this, since 1996, COPD mortality has fallen steadily in Galicia in both genders, without variations in the man-to-woman mortality ratio over time [8]. Our results are also in line with previous research finding that COPD mortality rates increase with increasing age [2].

Another interesting finding of this study is that the joinpoint analysis highlighted a recent, unexpected change in trends since the mid-2010s in men aged 80-84 years and 85+ years, with an acceleration of declining rates. In women aged 50 years and older, after a previous period of significant decline, rates have stabilized (50-54 age group), slowed their rate of decline (75-79 and 80-84 age groups) or even increased significantly (55-59, 60-64, 65-69 and 70-74 age groups) since the late 1990s and early 2000s.

It is well established that smoking is the leading cause and most important risk factor for developing COPD in Spain. Although it would be reasonable to interpret the observed overall decline in COPD mortality as confirming that public health interventions on smoking [13] and medical treatment have improved outcomes [14], the possible causes for the observed disparities between genders and age groups are of concern, and further studies are needed. A possible factor to take into account relates to changes in the prevalence of COPD in Spain. The EPI-SCAN II study recently analyzed prevalence figures with a broad geographic representation in the country [15]. The data from EPI-SCAN II indicate that the prevalence of women is increasing compared to men, and that in Madrid and Zaragoza the prevalence is currently higher in women. Given the narrowing of the gender gap that this entails, it can be expected that the mortality figures will show a similar trend.

The increase in the truncated rates in women is striking and contradicts previous analyses in the country [3,7,9,16,17]. This could be a consolidated trend leading to an exponential increase in mortality in women, as has already occurred in other tobaccorelated diseases such as lung cancer [18], and it will therefore be necessary to monitor this trend in the future. Various studies have revealed differences between men and women in the clinical consequences of COPD. In this context, some authors have pointed out a different symptomatic impact between the genders of a quantitative and qualitative nature, including fatigue, frailty, and poor sleep quality. Symptom management strategies should therefore address anxiety and depression more closely in women patients, while chest tightness or dyspnea should be monitored in men patients [19]. In addition, a number of biological differences can be observed between men and women with COPD, including differences in inflammatory and metabolic pathways related to obesity and fat distribution, immune cell function and autophagy, and the extent and distribution of emphysema and airway wall remodeling [20]. Although women with COPD experience higher levels of dyspnea and anxiety than men at comparable levels of age and forced expiratory volume in the first second, these differences do not translate into variations in mortality rates [21].

Our analysis has several limitations. First, the validity of the COPD codes recorded on death certificates is unknown. Although different authors have used various approaches to identify COPD cases in databases [22], the use of death certificate codes remains the most common method, thus making our data easier to compare with previous initiatives. Second, the study period spanned several ICD transitions, and may therefore have been subject to sudden artificial changes in mortality rates, such as when the change from ICD-9 to ICD-10 took place in Spain in 1999. However, there is no evidence of major changes associated with successive ICD revisions [23], and good concordance between ICD-9 and ICD-10 has been observed for the main causes of death and premature mortality in Spain [24]. Nevertheless, our definition of COPD mortality was deliberately broad, including all chronic lower respiratory tract causes, whether bronchitis or obstructive, with the sole exception of asthma. Narrower definitions are more prone to misclassification and to local variations due to medical practice or methods of coding deaths. Third, as in other mortality studies, we examined only COPD as the underlying cause of death. Studies using multiple causes of death provide a fuller picture of the burden associated with COPD [8]. In specific cases of chronic diseases, the assumption of a single underlying cause of death is often unrealistic, and the professionals responsible for certifying the cause of death often have limited information available when determining the underlying cause of death, which limits the choice of code to assign. The steady increase in the average age of patients dying from COPD in Spain (from 74 years for men and 77 years for women in 1980 to 82 years for men and 84 years for women in 2020) makes it difficult to determine the cause of death solely from these codes [25]. Fourth, our work is subject to the inherent limitations of all ecological studies. While this type of approach is useful for generating hypotheses, the lack of information on the distribution of possible risk and/or prognostic factors makes it difficult to verify these hypotheses at the patient level. Finally, our data were derived from information obtained from death certificates. Currently, the validity of death certificates in COPD cases is an issue of lively debate [7]. Several studies have shown underestimations of COPD deaths on death certificates [26], especially when the final cause of death is unclear. Data from the TORCH [27] trial confirmed that death certificates do not mention COPD in 42% of deaths of COPD patients, COPD is not mentioned anywhere on the death certificate in approximately 20% of these deaths, and, alarmingly, in 1 in 3 deaths caused by COPD exacerbation, COPD was not listed as the main cause of death. These findings suggest that our results may have underestimated mortality due to this cause of death.

In conclusion, our study showed age and gender differences in COPD mortality trends in Spain. Our data show the progression of mortality until 2020 with a newly described increase for women in truncated rates. The possible causes of the disparities observed between genders and age groups are of concern and require further study. Therefore, health authorities should be aware of this changing trend in women so that they are alert to the need to implement public health measures aimed at changing this trend.

## DATA AVAILABILITY

The data that support the findings of this study are derived from the following resources available in the public domain: Spanish National Institute of Statistics, available at https://www.ine.es/.

## Figures and Tables

**Table 1.** Age-specific and age-standardized mortality rates for men and women, 1980-2020

Variables			Age-specific rates^1^		Average APC	Trend 1		Trend 2		Trend 3		Trend 4	
			1980	2020	1980-2020	Period	APC	Period	APC	Period	APC	Period	APC
Men													
	Age (yr)												
		35-39	0.72	0.12	-3.7^*^	1980-1986	8.3	1986-2020	-5.6^*^	-	-	-	-
		40-44	1.47	0.51	-4.8^*^	1980-2000	-1.4	2000-2020	-8.0^*^	-	-	-	-
		45-49	3.49	1.47	-2.0^*^	1980-1984	15.0	1984-2020	-3.8^*^	-	-	-	-
		50-54	11.53	3.54	-2.5^*^	1980-1990	1.7	1990-2020	-3.8^*^	-	-	-	-
		55-59	17.93	8.97	-2.1^*^	1980-1990	4.0^*^	1990-2020	-4.1^*^	-	-	-	-
		60-64	45.69	21.32	-1.8^*^	1980-1991	4.0^*^	1991-2020	-3.9^*^	-	-	-	-
		65-69	75.28	45.44	-1.4^*^	1980-1991	5.5^*^	1991-2020	-3.9^*^	-	-	-	-
		70-74	152.87	84.45	-1.3^*^	1980-1990	6.0^*^	1990-1998	-1.8	1998-2020	-4.4^*^	-	-
		75-79	284.22	163.30	-0.9^*^	1980-1985	9.9^*^	1985-1997	1.9^*^	1997-2020	-4.5^*^	-	-
		80-84	452.32	317.03	-0.4	1980-1988	7.9	1988-1999	1.1	1999-2017	-3.9^*^	2017-2020	-9.8^*^
		≥85	791.47	732.55	0.0	1980-1989	6.7^*^	1989-1999	2.5^*^	1999-2015	-2.7^*^	2015-2020	-7.6^*^
	Age-standardized rates^1^												
		All ages	18.02	11.48	-0.9^*^	1980-1989	6.1^*^	1989-1999	-0.2	1999-2020	-4.0^*^	-	-
		35-64	11.17	4.89	-2.1^*^	1980-1990	4.0^*^	1990-2020	-4.0^*^	-	-	-	-
Women													
	Age (yr)												
		35-39	0.45	0.06	-5.1^*^	1980-2020	-5.1^*^	-	-	-	-	-	-
		40-44	0.38	0.26	-3.0^*^	1980-2020	-3.0^*^	-	-	-	-	-	-
		45-49	1.08	0.41	-2.5^*^	1980-2020	-2.5^*^	-	-	-	-	-	-
		50-54	1.49	1.25	0.1	1980-1987	9.7^*^	1987-1994	-10.1^*^	1994-2020	0.6	-	-
		55-59	3.17	4.06	0.4	1980-1990	4.6^*^	1990-2001	-7.8^*^	2001-2020	3.2^*^	-	-
		60-64	8.04	8.45	0.5	1980-1990	1.6	1990-2007	-4.6^*^	2007-2020	6.8^*^	-	-
		65-69	18.80	17.02	-0.3	1980-1987	3.5^*^	1987-2010	-4.6^*^	2010-2020	7.4^*^	-	-
		70-74	42.52	18.45	-1.5^*^	1980-1989	2.6^*^	1989-1999	-3.6^*^	1999-2007	-8.3^*^	2007-2020	1.6^*^
		75-79	80.25	29.90	-2.3^*^	1980-1987	4.0^*^	1987-1998	-1.9^*^	1998-2008	-7.6^*^	2008-2020	-1.6^*^
		80-84	173.14	55.06	-2.8^*^	1980-1989	2.4^*^	1989-1999	-1.6^*^	1999-2006	-8.2^*^	2006-2020	-3.9^*^
		≥85	355.06	151.99	-2.1^*^	1980-1999	0.8^*^	1999-2020	-4.6^*^	-	-	-	-
	Age-standardized rates^1^												
		All ages	5.71	2.82	-1.5^*^	1980-1986	3.7^*^	1986-1999	-1.5^*^	1999-2007	-6.3^*^	2007-2020	-0.9^*^
		35-64	2.06	1.96	0.1	1980-1986	4.6^*^	1986-2006	-3.8^*^	2006-2020	3.8^*^		

APC, annual percent change.

1 Expressed as rates per 100,000 person-years.

* p<0.05.
